# Reduced Selenium-Binding Protein 1 in Breast Cancer Correlates with Poor Survival and Resistance to the Anti-Proliferative Effects of Selenium

**DOI:** 10.1371/journal.pone.0063702

**Published:** 2013-05-21

**Authors:** Sheng Zhang, Feng Li, Mamoun Younes, Hao Liu, Changyi Chen, Qizhi Yao

**Affiliations:** 1 Molecular Surgeon Research Center, Michael E. DeBakey Department of Surgery, Baylor College of Medicine, Houston, Texas, United States of America; 2 Department of Molecular Virology and Microbiology, Baylor College of Medicine, Houston, Texas, United States of America; 3 Department of Pathology and Laboratory Medicine, University of Texas Medical School at Houston, Houston, Texas, United States of America; 4 Division of Biostatistics, Dan L. Duncan Cancer Center, Baylor College of Medicine, Houston, Texas, United States of America; Sun Yat-sen University Medical School, China

## Abstract

Supplemental dietary selenium is associated with reduced incidence of many cancers. The antitumor function of selenium is thought to be mediated through selenium-binding protein 1 (SELENBP1). However, the significance of SELENBP1 expression in breast cancer is still largely unknown. A total of 95 normal and tumor tissues assay and 12 breast cancer cell lines were used in this study. We found that SELENBP1 expression in breast cancer tissues is reduced compared to normal control. Low SELENBP1 expression in ER^+^ breast cancer patients was significantly associated with poor survival (*p*<0.01), and SELENBP1 levels progressively decreased with advancing clinical stages of breast cancer. 17-β estradiol (E2) treatment of high SELENBP1-expressing ER^+^ cell lines led to a down-regulation of SELENBP1, a result that did not occur in ER^–^ cell lines. However, after ectopic expression of ER in an originally ER^–^ cell line, down-regulation of SELENBP1 upon E2 treatment was observed. In addition, selenium treatment resulted in reduced cell proliferation in endogenous SELENBP1 high cells; however, after knocking-down SELENBP1, we observed no significant reduction in cell proliferation. Similarly, selenium has no effect on inhibition of cell proliferation in low endogenous SELENBP1 cells, but the inhibitory effect is regained following ectopic SELENBP1 expression. Furthermore, E2 treatment of an ER silenced high endogenous SELENBP1 expressing cell line showed no abolishment of cell proliferation inhibition upon selenium treatment. These data indicate that SELENBP1 expression is regulated via estrogen and that the cell proliferation inhibition effect of selenium treatment is dependent on the high level of SELENBP1 expression. Therefore, the expression level of SELENBP1 could be an important marker for predicting survival and effectiveness of selenium supplementation in breast cancer. This is the first study to reveal the importance of monitoring SELENBP1 expression as a potential biomarker in contributing to breast cancer prevention and treatment.

## Introduction

Breast cancer is the most frequently diagnosed cancer and the leading cause of cancer death among women in the United States [1]. Approximately 75–80% of breast cancers express the estrogen receptor (ER), which can be targeted by selective estrogen receptor modulators such as tamoxifen or aromatase inhibitors to block estrogen action. Nevertheless, approximately 40% of patients fail to respond to current treatment strategies for breast cancer and ultimately die from the disease. Therefore, identification of more reliable markers to predict the effectiveness of specific therapies and understanding of the respective molecular mechanisms that enhance treatment efficacy are urgently needed.

Selenium (Se) is a trace element that is essential to many biological processes and possesses anti-carcinogenic properties. Supplemental dietary Se was first observed to play a role in reducing cancer risk over forty years ago [2]. Se has since been associated with significant reductions of 39% to 52% in total incidence and mortality of various cancers, including prostate, lung, pancreatic, and colorectal cancers [3], [4]. A number of potential mechanisms have been proposed for the preventative effects of Se, including stimulation of apoptosis, induction of cell-cycle arrest, inhibition of tumor cell invasion, and influences on estrogen and androgen-receptor expression [5]–[7].

The physiological function of Se related to its anticancer effects is associated with selenium containing proteins [8], which can be categorized into three groups [9]: specific selenoproteins (which contain Se in the form of selenocysteine incorporated into the polypeptide chain), nonspecific selenium-containing proteins (Se is incorporated nonspecifically into proteins), and selenium-binding proteins (selenium is only attached to the molecules), which include liver fatty acid-binding protein, protein disulfide isomerase, and Selenium-binding protein 1 (SELENBP1, also called SBP1, hsP56), respectively. The SELENBP1 gene is located at chromosome 1q21–22; the mRNA sequence of the gene is composed of 1721 nucleotides encoding 640 amino acids. SELENBP1 mRNA is abundantly expressed in many normal human tissues [10], [11], while several groups have recently reported significantly decreased SELENBP1 protein levels in many types of cancers including lung [12], [13], gastric [14], ovarian [15], colorectal [16] and thyroid [17], compared to normal tissue. SELENBP1 is implicated in cell-growth regulation [18], [19]. Low SELENBP1 expression levels are associated with poor patient prognosis [12], [15], [16], [20], [21], clearly suggesting a role for SELENBP1 in regulating the growth and progression of cancer. Nevertheless, the biological functions SELENBP1 plays in breast cancer have not been explored.

Almost all breast cancer patients are female and usually have persistently elevated estrogen levels in the blood, which are consistently associated with breast cancer risk [22]. As such, a hormonal effect must be considered in any study of breast cancer. The growth of breast cancer cells is known to be regulated by estrogen through binding to ER, which affects cell growth by inducing cell proliferation [23], [24] and preventing apoptotic cell death [25], [26]. Among estrogen-responsive genes found in previous studies, mRNA levels of SELENBP1, but not other selenium-containing proteins, were found to be down-regulated by estrogen treatment in breast cancer cells [27], [28]. Combined with controversial results in females from prospective studies on Se, we hypothesize that there is a connection between estrogen, ER, and SELENBP1, which could address the unique role of SELENBP1 in the pathogenesis and prevention of breast cancer. In this study, we determined the levels of SELENBP1 expression in breast cancer tissue arrays; we show the correlation of SELENBP1 expression with other known breast cancer markers as well as uncover a correlation between SELENBP1 expression and patient survival. In addition, we determined that estrogen is responsible for the regulation of SELENBP1, and we demonstrate the functional role of SELENBP1 in the proliferation of breast cancer cell lines with Se-treatment.

## Materials and Methods

### Cells, Plasmids, Chemicals, and Antibodies

Breast cancer cell lines MB231, MB453, MB468, HS578T, T47D, ZR75B, HCC70, HCC1937, MCF7, MCF10A, BT474, and SKBR3 cells, were a kind gift from Dr. Yi Li (Baylor college of Medicine) who originally obtained these cells lines from ATCC and Dr. Neve’s lab, all cells were characterized and maintained as previously described [29]. To maintain MCF10A cells, 20 ng/ml EGF, 100 ng/ml cholera toxin, 0.01 mg/ml insulin, and 500 ng/ml hydrocortisone (Sigma) were added to the media. Charcoal-stripped fetal bovine serum (FBS) and horse serum (HS) were purchased from Invitrogen and HyClone. SELENBP1 expression plasmid was obtained from GeneCopoeia (Rockville, MD). ER**α**-specific siRNA duplexes and SELENBP1specific shRNA was obtained from OriGene (Rockville, MD). ER plasmid is a kind gift from Dr. Susan Fuqua (Baylor College of Medicine). MTT and anti–β-actin antibody were purchased from Sigma. Anti-SELENBP1 antibody was purchased from MBL International. Goat anti-rabbit and goat anti-mouse IgG (H&L) antibody–horseradish peroxidase were obtained from Cell Signaling. Anti-ERα antibody was purchased from Vector Laboratories (Burlingame, CA).

### Breast Cancer Tissue Array Slides

Three sets of breast cancer tissue arrays (including normal, primary tumor, and metastases) were obtained from Imgenex or Biomax Inc. Both companies stated that each specimen collected from any clinic was consented to by both hospital and individual. Discrete legal consent form was obtained and the rights to hold research uses for any purpose or further commercialized uses were waived. A total of 21 normal and 74 tumor tissues from female patients ranging in age from 28 to 84 years (47.2±9.37) were analyzed.

### Immunohistochemistry (IHC)

Standard immunohistochemistry staining of breast cancer tissue arrays was performed utilizing a Dako autostainer (Dako, Carpinteria, CA). Briefly, the arrays were de-paraffinized and rehydrated through series of xylenes and graded alcohols ending in BBS. Steam heat antigen retrieval was carried out in 10 mM sodium citrate buffer (pH 6) for 20 min followed by cooling off at room temperature for 15 minutes. Slides were then incubated with 1∶100 dilution of the anti-human SELENBP1 antibody in Dako antibody diluent for 30 minutes at room temperature and the bound antibody was detected using Dako Envision plus Mouse Peroxidase kit with DAB as chromogen. Slides were counterstained with hematoxylin, dehydrated, and coverslipped.

### IHC Scoring

IHC scoring was performed on the tissue microarrays according to the Allred method [30]. Briefly, each sample was given a proportion score: 0: negative; 1∶0∼1/100 cells staining; 2∶1/100–1/10; 3∶1/10–1/3; 4∶1/3–2/3; 5∶2/3–3/3 cells staining; and an intensity score: 0: negative; 1: weak; 2: intermediate; 3: strong. The total score (TS) is the proportion score plus intensity score. The optimal cut-off values for the Allred score were determined based on data we obtained from the tissue arrays.

### Immunoblotting Analysis

Plasmids were transfected by using lipofectamine 2000 according to manufacture’s recommendation. Protein expression levels were determined by western blot as described previously [31]. Briefly, cells were lysed with 100 µL of RIPA lysis buffer (Sigma) with protease inhibitors cocktail (Roche). The lysates containing 50 µg total protein were loaded and separated using SDS-PAGE and then transferred to a PVDF membrane (Bio-Rad Laboratories). The levels of SELENBP1 and β-actin were detected by using specific primary antibodies. 1∶1000 dilution of SELENBP1 or 1∶2000 of ERα Ab was used. Quantitation of protein bands was done using ImageJ software from NIH. Briefly, each protein band was scanned at a resolution of a least 600 dpi and the density of protein bands was measured. The expression level of each band was normalized to loading control. The relative expression level of a target protein was calculated by being normalized to the protein in the untreated control which was designated to be 100%.

### Quantitative PCR

The SELENBP1 mRNA was analyzed using real-time RT-PCR as described previously (Li et al., 2008). Briefly, total RNA was extracted by using RNAqueous-4PCR kit. Real-time RT-PCR was performed with total RNA by using a SYBR supermix kit from Bio-Rad. The PCR reaction included the following components: 0.1 µM each primer, 2 µl cDNA templates, and diluted 2X iQ SYBR green supermix, for 35 cycles at 95°C for 20 sec and 60°C for 1 min. PCR efficiency was pre-examined by serially diluting the template cDNA, and the melting-curve data were collected to check the specificity of amplification. Each cDNA sample was run as triplicates, and a corresponding no-reverse transcriptase (RT) mRNA sample was also included as a control. The human β-actin primer was included in each sample to avoid variations. The mRNA level of each gene was normalized to that of the β-actin mRNA. The amount of PCR products was measured of threshold cycle (Ct) values. The relative mRNA level was presented as unit values of 2∧[Ct(β-actin) − Ct(gene of interest)]. The primers used for SELENBP1 and β-actin were as follows: SELENBP1-F (5′-ATGTGGGAATTGTGGACCCG-3′), SELENBP1-R (5′-TGCCTGTGTTTCGGTAAATGC-3′), β-Actin-F (5′-CATGTACGTTGCTATCCAGGC-3′), and β-Actin-R (5′-CTCCTTAATGTCACGCACGAT-3′).

### Treatments with Estrogen or Selenium

ER^+^ cell lines MCF-7 and T47D, or ER^–^ cell lines SK-Br-3 and MB453, or ER transfected SK-Br-3 (SKBr-3 ER^+^) and MB453 (MB453 ER^+^) cells were seeded into 6-well plates. After the cells reached ∼50% confluence, the medium was replaced with the same growth medium containing 10 nM 17β-estradiol (E2) or methylseleninic acid (MSA) in the presence of 10% charcoal-stripped FBS at the indicated concentrations. The cells were collected at different time points. Whole-cell lysates were prepared according to procedures already described and used in a Western blot.

### Cell Proliferation Measurement by Cell Viability Assay (MTT)

The effect of selenium on cell proliferation was determined using the 3-(4,5-Dimethyl-2-thiazolyl)-2,5-diphenyl-2H-tetrazolium bromide (MTT) assay to measure cell viability. Briefly, 2,000 SELENBP1 high expressing cells (MCF7 vector control and MB231 with SELENBP1 transfected cells) or SELENBP1 low expressing cells (MCF7 with SELENBP1-specific shRNA transfected cells or MB231 vector control) cells were plated and serum-starved for 24 h. A day 0 control reading (viability corresponding to basal number of cells plated) was then measured by MTT. Medium with different concentrations of MSA was added to each well and incubated for 3 days. Cell viability was measured using MTT as follows: 1 mg/mL of MTT in medium with 2% serum was added to each well and incubated for 2 h at 37°C and 5% CO_2_. An extraction buffer (20% SDS, 50% dimethylformamide) was added, following an overnight incubation. Absorbance was measured at 570 nm using a 96-well multi-scanner (EL-800 universal microplate reader; BioTek, Inc.). The proliferation capacity of the cells was measured by dividing the viability of MSA treatment cells by the viability of untreated control cells.

### Statistical Analysis

Pathology scoring results were summarized and plotted using mean ± SD. Comparisons between two or more groups were analyzed using T test or ANOVA, respectively. Survival curves were estimated and plotted by the Kaplan-Meier method. Log-Rank test was used to compare the survival difference between patients with high or low SELENBP1 expression (cut-off = 7). A *P*-values <0.05 was considered statistically significant. Quantitative results are shown as mean+SD. The statistical analysis was done by Student’s t test for paired data between control and treated groups.

## Results

### SELENBP1 Expression is Reduced in Human Breast Cancer Tissues

**Figure 1:**
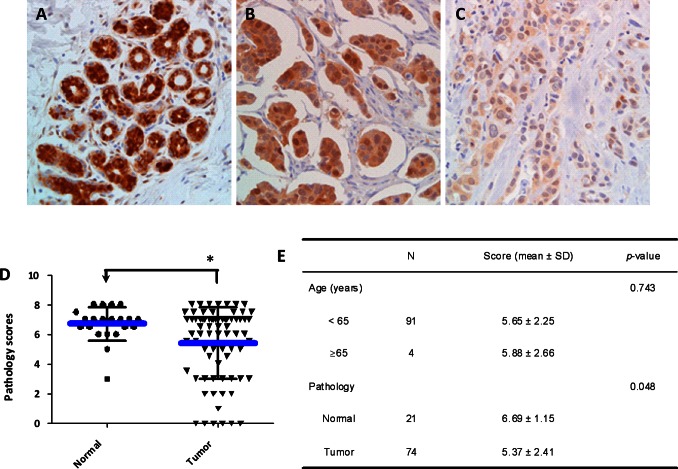
The Expression of SELENBP1 in Normal and Tumor Breast Tissues. Breast cancer tissue arrays were stained by immunohistochemistry using anti-human SELENBP1 antibody at 1∶100 dilution. Positive stained cells are shown in dark brown color. (A) Strong positive staining of SELENBP1 in normal breast tissue under low power view (200X). (B–C) Weak positive to negative staining of SELENBP1 in breast cancer tissues under high power view (400X). (D) The Allred scoring distributions of SELENBP1 expression in normal and tumor tissue groups. Inside lines represent means and standard deviations. *p<0.05. (E) Statistical results for the difference between normal and tumor tissues as analyzed by Kruskal-Wallis test.

To determine SELENBP1 expression levels in breast cancer tissues, immunohistochemical staining of SELENBP1 in 3 sets of formalin-fixed tissue arrays (US Biomax and Imgenex Inc.) was performed. As shown in Fig. 1A, positive staining of SELENBP1 was observed in normal breast ducts and lobular units, while low to negative staining was seen in tumor tissues in Fig. 1B (invasive ductal carcinoma showing micropapillary morphology) and in Fig. 1C (poorly differentiated invasive ductal carcinoma, high nuclear grade). Overall, the Allred score of SELENBP1 expression in normal tissues was 6.69±1.15, with a statistically significant reduction in SELENBP1 expression of 5.37±2.41 in the tumor tissue group (*p*<0.05, Fig. 1D and E). In supporting our results, re-analysis of microarray raw data from NCBI database (GEO: GSE7377) also showed that the expressions of SELENBP1 in hyperplastic enlarged lobular cells of breast tissues by laser capture microdissection (LCM) were significantly lower than that in normal terminal duct lobular cells (Figure S1 in File S1). We found no statistically significant correlation between SELENBP1 expression and patient age ≥65 (5.88±2.66) and <65 (5.65±2.25) (Fig. 1E). Therefore, this result indicates that SELENBP1 expression is reduced in breast cancer tissue and it is not correlated with age.

### Gradual Reduction of SELENBP1 Expression through Breast Cancer Progression

**Figure 2:**
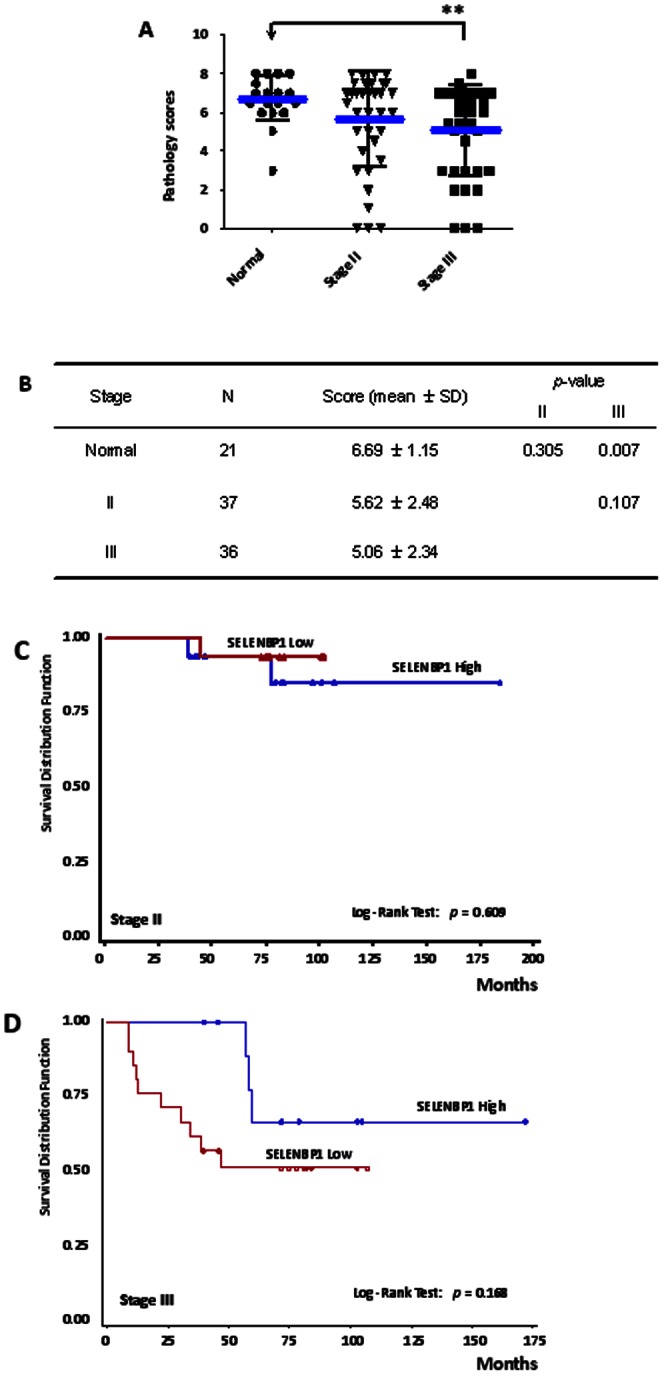
SELENBP1 Expression is Progressively Reduced in Advancing Clinical Stages in Breast Cancer Tissues. (A) The scoring distributions of SELENBP1 expression in normal tissues and tumor tissues at stage II and stage III. Inside lines represent means and standard deviations. **p<0.01. (B) Statistical results for the difference between normal and tumor tissues as analyzed by Kruskal-Wallis test. (C) Survival curves of breast cancer patients with respect to different SELENBP1 expression levels are shown at stage II and (D) stage III. Blue and red lines represent the SELENBP1-high and SELENBP1-low groups, respectively.

To determine whether the level of SELENBP1 expression was correlated with disease stage, differential SELENBP1 expression in stages II and III was compared with each other and with the normal group in the same tissue arrays. We found that patients in stage III of breast cancer had the biggest decrease of SELENBP1 expression (5.06±2.34) among the three groups (Fig. 2A). Compared to normal tissues (6.69±1.15), this decrease was statistically significant (*p* = 0.007) (Fig. 2B). There was a trend towards decreased expression of SELENBP1 from stage II (5.62±2.48) to stage III (5.06±2.34), but it did not reach the statistical significance due to limited sample size. There was also no significant difference in survival in stage II patients based on differential levels of SELENBP1 (Fig. 2C). However, within the patients in stage III, higher SELENBP1 expression tended to correlate with longer survival than lower expression group (*p* = 0.168, log-ranked test) (Fig. 2D), though this failed to reach statistical significance, again likely due to limited sample size. This indicates that reduced SELENBP1 expression may correlate with tumor progression in later stages in the pathogenesis of breast cancer, as is the case with colorectal cancer [16].

### Higher SELENBP1 in ER^+^ Patients Predicts Better Survival

**Figure 3:**
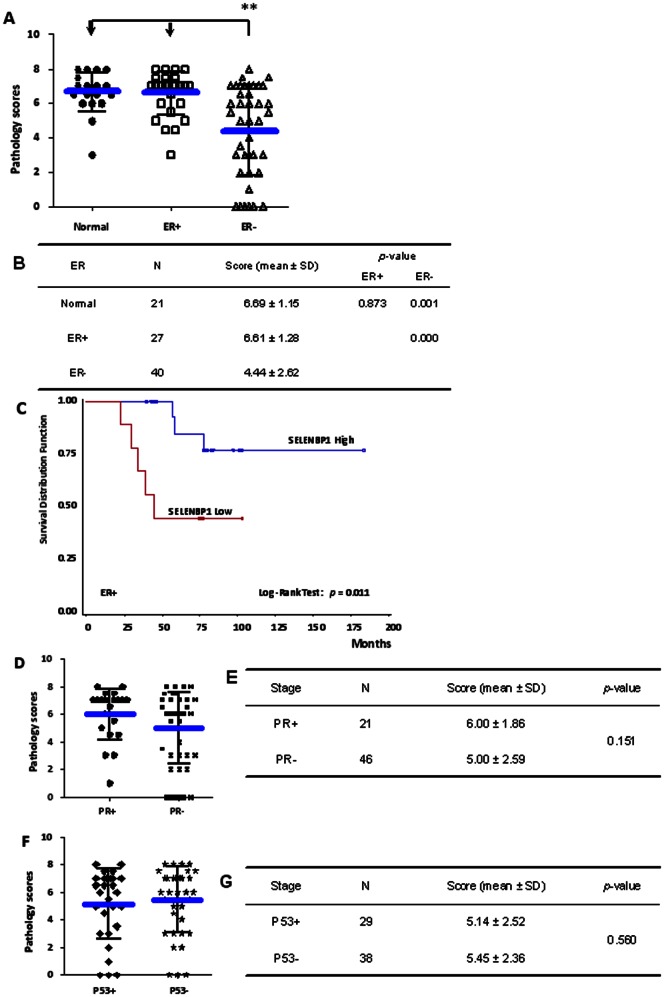
The Correlation of SELENBP1 Expression with ER, PR, and TP53 in Breast Cancer Tissues. (A) The scoring distributions of SELENBP1 expression in normal tissues and tumor tissues with ER^+^ and ER^–^ status. The inside lines represent means and standard deviations. **p<0.01. The difference between normal and ER^+^ and ER^–^ tumor tissues was analyzed by Kruskal-Wallis test and statistical results are shown (B). Survival curves of breast cancer patients with respect to different SELENBP1 expression are shown in ER^+^ group in (C). The blue line is the SELENBP1-high group and the red line is the SELENBP1-low group. The scoring distributions of SELENBP1 expression in normal and tumor tissues with PR^+^/PR^–^ are shown in (D) and TP53^+^/TP53^−^ shown in (F). The difference between PR^+^/PR^–^ and TP53^+^/TP53^−^ tumor tissues was analyzed by Kruskal-Wallis test and statistical results are shown in (E) and (G), respectively.

To clarify whether levels of SELENBP1 expression are associated with ER status in breast cancer, we analyzed SELENBP1 expression levels with respect to ER^+^ and ER^–^ samples as well as the patient survival within the ER^+^ patients. As shown in Fig. 3A, the expression level of SELENBP1 in ER^–^ breast cancer tissues was significantly lower than in ER^+^ tissues (4.44±2.62 versus 6.61±1.28, *p* = 0.000) (Fig. 3A and B). Moreover, within ER^+^ breast cancer patients, higher SELENBP1 expression was significantly associated with better survival than lower expression (*p* = 0.011, log-ranked test) (Fig. 3C). These results indicate that ER^–^ patients express lower levels of SELENBP1 compared to ER^+^ patients, and higher SELENBP1 in ER^+^ patients predicts better prognosis.

In addition, we also analyzed the SELENBP1 level with progesterone receptor (PR) and TP53 status in the tissue array. However, we found no significant difference in SELENBP1 expression with respect to PR expression status (Fig. 3D and E) or TP53 mutation status (Fig. 3F and G).

### SELENBP1 is Differentially Expressed in Human Breast Cancer Cell Lines

**Figure 4:**
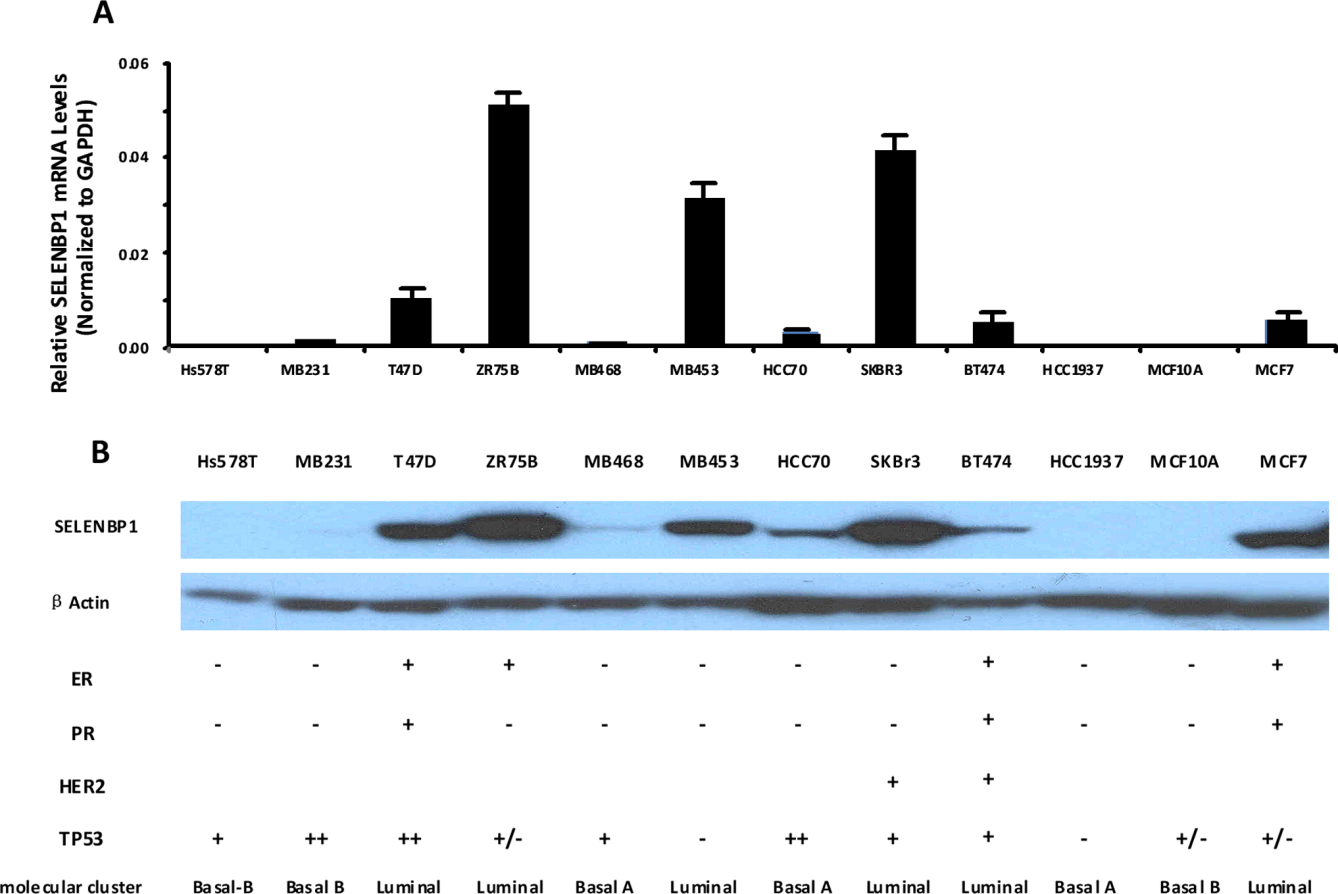
Differential SELENBP1 Expression in 12 Common Breast Cancer Cell Lines. (A) The SELENBP1 mRNA expression levels were determined by using quantitative real-time PCR. (B) The SELENBP1 protein expression levels were determined by western blotting. ER status of the cell lines were labeled according to published literatures.

We examined SELENBP1 expression levels in a panel of breast cancer cell lines which included 4 ER^+^ (MCF7, BT474, ZR75B, and T47D) and 7 ER^–^ cell lines (HCC1937, SKBR3, HCC70, MB453, MB468, MB231, HS578T). Based on the molecular classification of breast cancer cell lines [29], they can be classified into luminal and basal-like subtypes as found in primary tumors. The basal-like cell lines resolve further into two distinctive clusters (Basal A and Basal B). Therefore, these cell lines we used are 3 basal A (MB468, HCC70, HCC1937), 2 basal B (HS578T, MB231), and 6 luminal cell lines (T47D, ZR75B, MB453, SKBR3, BT474, MCF7). The relative SELENBP1 mRNA levels normalized to GAPDH in each cell lines are shown in Fig. 4A. The SELENBP1 protein expression levels are shown in Fig. 4B. Generally, most of the ER^–^ breast cancer cell lines were found to express little to no SELENBP1 when compared with ER^+^ breast cancer cell lines. While among the ER^+^ breast cancer cell lines, varying levels of SELENBP1 expression were found. Besides ER status, SELENBP1 expression also was found to be associated with cell molecular subtype. All 6 luminal type breast cancer cells expressed certain levels of SELENBP1. 2 basal B and 2 basal A breast cancer cells were found to have no SELENBP1 expression, while 1 basal A breast cancer cell line, HCC70 cell, had low SELENBP level. Re-analysis of microarray raw data from NCBI database (GEO: GSE12777) and EBI database (ARRAYEXPRESS: E-TABM-157) showed similar results as above (Figure S2 in File S1). This may indicate that SELENBP1 is mostly expressed in ER^+^ and luminal breast cancer cells but not ER^–^ and basal breast cancer cells which are more aggressive cell lines.

### Estrogen Treatment Down-regulates SELENBP1 Protein

**Figure 5:**
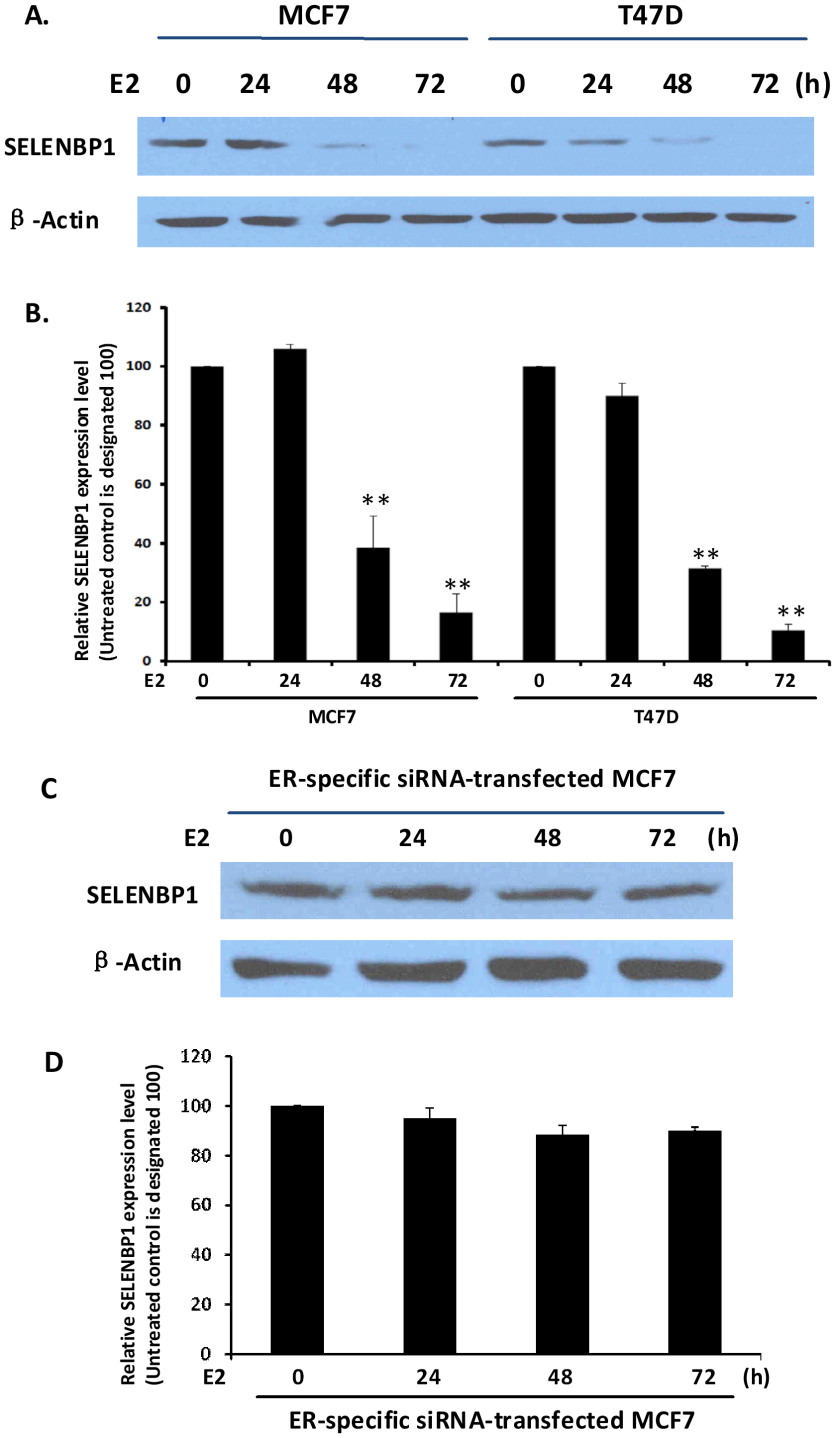
SELENBP1 Expression is Reduced in Response to estrogen Treatment. (A–B) MCF7 and T47D ER+ cells; (C–D) MCF7 cells with ER knock-down; (E–F) SKBr3 and MB453 ER^–^ cells; and (G–H) ER transfected SKBr3 and MB453 cells were cultivated in complete DMEM media containing 10% charcoal-stripped FBS. E2 at 10 nM concentration was added to the cell. Cell lysates were collected at different time points and SELENBP1 expression was shown in western blot with anti-SELENBP1 antibody at a 1∶1000 dilution. B-actin was used as a loading control. (B, D, F, and H) showed quantitation of protein expression levels in relation to no E2-treated protein level. Results are representatives of at least two independent experiments. (*p<0.05, **p<0.01).

Based on the results of screening of 12 breast cancer cell lines, we chose MCF7, T47D, SKBR3 and MB453 cells as representatives of different ER status in breast cancer cells but all have high SELENBP1 expression for further studies. To determine whether estrogen can modulate SELENBP1 expression in ER^+^ (MCF7 and T47D cells) and ER^–^ (SKBR3 and MB453 cells) breast cancer cells, we treated the cells with 17β-estrodial (E2) in complete media containing charcoal-stripped FBS for different length of times. As shown in Fig. 5A, SELENBP1 expression in ER^+^ MCF-7 and T47D cells decreased with E2 treatment in a time-dependent manner. After quantitation of SELENBP1 expression proteins bands shown in Fig. 5B, we found a statistically significant reduction of SELENBP1 expression starting 48 h of E2 treatment in both ER^+^ cell lines. To further determine if E2 regulates SELENBP1 expression via ER, we used ERα-specific siRNA strategy to silence ERα expression. The successful silencing of ERα in MCF7 cells is shown in Figure S3 in File S1. As shown in Fig. 5C and 5D, there was no SELNBP1 reduction upon E2 treatment in the MCF7 ER silenced cells. Similarly, there was also no difference between the E2 treated and untreated ER^–^ SKBR3 and MB453 cells (Fig. 5E, F) in SELENBP1 expression levels. Re-analysis of microarray raw data from NCBI database (GEO: GSE11324 and GSE11352) also demonstrated gradual decrease of SELENBP1 expression in MCF7 cells with E2 treatment in a time-dependent manner compared with untreated controls (Figure S4 in File S1).

To further determine the action of estrogen through ER is indeed involved in regulation of SELENBP1 expression, we ectopically expressed ERα in ER^–^ cell lines SKBr3 and MD453 now designated as SKBr3 (ER^+^) and MD453 (ER^+^) cell lines. The expression of ER in these cell lines before and after the transfection was determined and confirmed by western blot as shown in Figure S5 in File S1. Upon treatment with E2, we observed a gradual and yet significant reduction of SELENBP1 expression in these ER^+^ cells, although less dramatically than in the native ER^+^ cells (Fig. 5G, H). This may be due to the fact that the levels of ER in these transfected cell lines are not comparable to the endogenous ER^+^ cell lines. However, this result also supports the conclusion that estrogen levels can affect SELENBP1 expression in ER^+^ breast cancer cells.

### SELENBP1 is Important in Conferring Inhibition of Cell Proliferation upon Se Treatment

**Figure 6:**
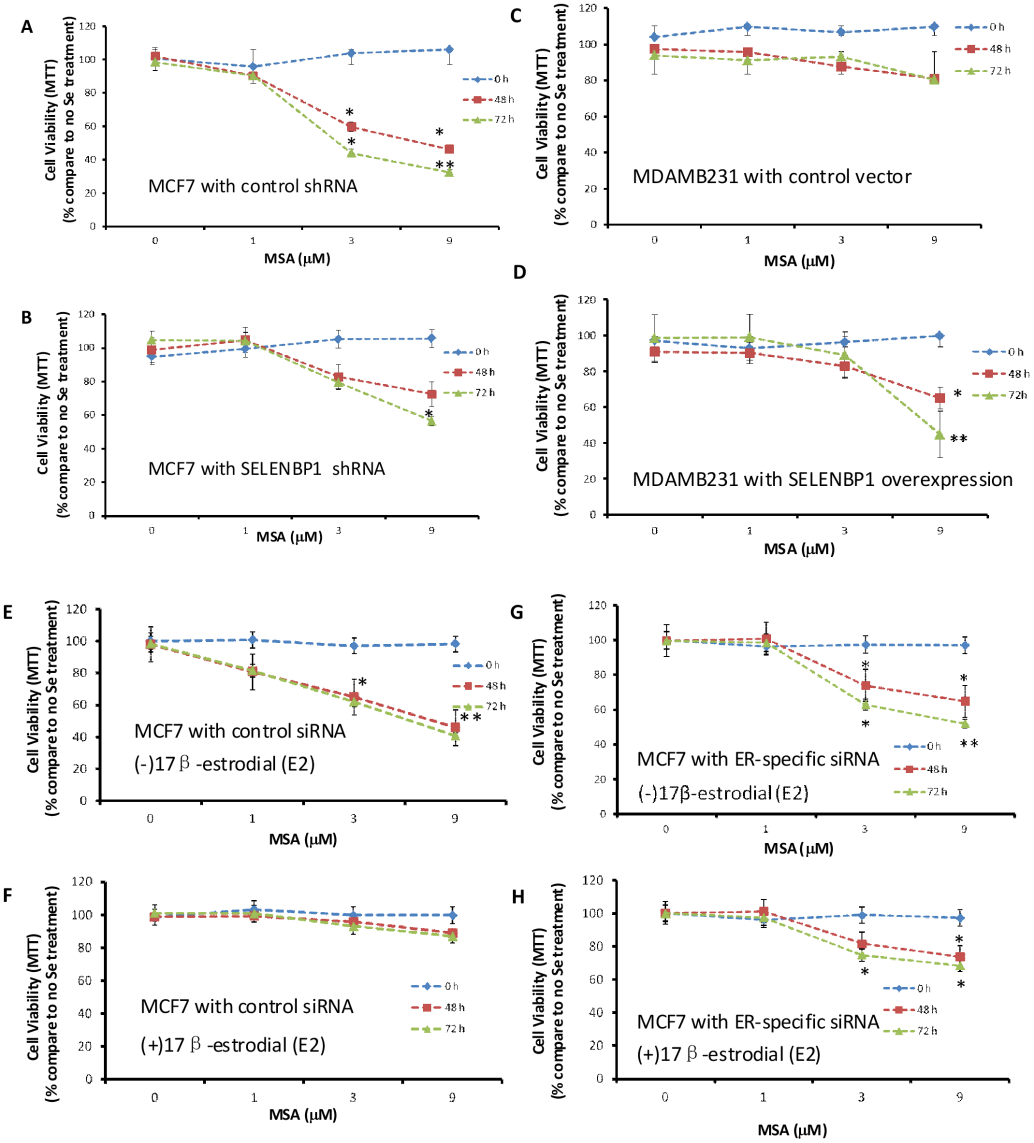
Cell Proliferation is Inhibited in SELENBP1 High Expression Cell Lines but not in SELENBP1 Low Expression Cell Lines upon MSA Treatment. Cells were cultivated in complete DMEM media containing 10% charcoal-stripped FBS. Different amounts of methylseleninic acid (MSA) were added to the cell in the cells with SELENBP1 high expression low expression levels. Cell viability was determined by MTT assay at 0, 48, and 72 h. The percentages of cell viability compared to no Se treatment control are plotted. (A). MCF7 transfected with vector control; (B). MCF7 transfected with shRNA to knock down SELENBP1; (C). MB231 transfected with vector control; (D). MB231 transfected with over-expressing SELENBP1. MCF7 transfected with control siRNA in the absence of E2 (E) and in the presence of E2 (F). MCF7 transfected with ERα specific siRNA in the absence of E2 (G) and in the presence of E2 (H). Results are representatives of at least two independent experiments. (*p<0.05).

We have shown above that estrogen can downregulate SELENBP1, now we want to determine whether SELENBP1 plays a key role in conferring the cell proliferation reduction upon Se treatment. Selenium-mediated inhibition of breast cancer growth was compared between the cell line with high endogenous SELENBP1 (MCF7 transfected with shRNA vector control) and SELENBP1 knock-down cells (MCF7 transfected with SELENBP1-specific shRNA). The successful knock-down of SELENBP1 is determined in the Figure S6 in File S1. Cells were treated with increasing concentrations of methylseleninic acid (MSA, an active form of selenium *in vitro*). The cell viability of MCF7 cells and MCF7 cells with SELENBP1 knock-down was determined by using MTT assay compared with the cell viability with no MSA treatment control. As shown in Fig. 6A, selenium inhibited growth of MCF7 cells in a dose-dependent manner at both 48 (*p*<0.05) and 72 h (p<0.01). A more than 50% inhibition of cell proliferation was observed when 9 µM of MSA was used. In contrast, when SELENBP1 was knocked down in Fig. 6B, no significant growth inhibition was observed even at the high dose of 9 µM of MSA. This result indicates that SELENBP1 plays an important role in conferring Se treated inhibition of cell proliferation.

Additionally, when we ectopically overexpress SELENBP1 in an originally SELENBP1 low MB231 cells (Confirmation of successful SELENBP1 overexpression is shown in Figure S6 in File S1), it restores the inhibition of cell growth activity at a high dose of MSA 9 µM (Fig. 6D) in the originally no response cells (Fig. 6C). These results further indicate that SELENBP1 protein level may influence the ability of selenium supplementation to inhibit breast cancer growth.

To further investigate whether E2-mediated changes in SELENBP1 protein influence the ability of Se to inhibit breast cancer growth, we used ERα specific siRNA to knock-down ER expression in MCF7 cells (shown in Figure S3 in File S1) and treated cells with E2 to compare the proliferation ability upon increasing concentrations of MSA treatment in ER^+^ and ER silenced MCF7 cells. As shown in Fig. 6E and F, MCF7 cells were transfected with control siRNA (universal scrambled negative control siRNA duplex), without E2 treatment which means intact high level of SELNBP1 expression, cell proliferation was inhibited in a dose-dependent manner at both 48 and 72 h (*p*<0.01) after MSA treatment (Fig. 6E). Approximately a 50% inhibition of cell proliferation was observed when 9 µM of MSA was used. In contrast, after E2 treatment which means downregulated SELENBP1 in the cells, MSA treatment has no significant growth inhibition effect even at the high dose of 9 µM of MSA (Fig. 6F). On the contrary, while in Fig. 6G and H, ER expression in MCF7 cells were silenced, even after E2 treatment, since SELENBP1 expression level was not affected by E2 treatment and kept high, we did not observe any significant abolished cell proliferation inhibition activity upon MSA treatment. These data indicate that estrogen-mediated changes in SELENBP1 protein can indeed influence the ability of SE to inhibit breast cancer growth.

## Discussion

In this study, we found that breast cancer tumor tissues had reduced levels of SELENBP1 expression compared to normal tissue, with a significant decrease of SELENBP1 was confirmed in late-stage (Stage III) tumors. Importantly, SELENBP1 expression was decreased in ER^–^ tumors but not ER^+^ tumors; however, low SELENBP1 expression in ER^+^ patients was associated with poor survival. In support of the clinical data, a similar expression pattern was observed for SELENBP1 in 12 commonly used breast cancer cell lines. SELENBP1 was generally expressed in ER^+^ and luminal-type cell lines but not ER^–^ and basal-type cell lines. In ER^+^ SELENBP1 high expressing breast cancer cells, addition of exogenous estrogen downregulated SELENBP1 expression, and knocking down SELENBP1 confers the cell resistance to selenium treated cell proliferation inhibition. In ER silenced SELENBP1 high expressing breast cancer cells, E2 treatment does not affect the cell proliferation inhibition ability of selenium treatment.

SELENBP1 has been reported to be down-regulated in many types of cancers [12]–[17], [32]–[34]. In our tissue array staining of breast cancer samples, the level of SELENBP1 expression was decreased in tumor tissues compared to normal, which is consistent with previous reports. The data from Wulfkuhle *et al*. shows that SELENBP1 was the most downregulated protein (more than 46 fold) in whole and/or laser capture microdissected tumor tissues of breast cancer patients by proteomic analysis in two-dimensional gel electrophoresis and MS sequencing [35]. Our tissue array also showed that SELENBP1 expression was dramatically reduced in stage III and ER^–^ tumor tissues. Studying estrogen receptor status in breast cancer, Gruvberger and collaborators also demonstrated that a high level of SELENBP1 expression was present in ER^+^ tumors but not ER^–^ ones (Fig. 1 and Table 3 in [36]). Thus, the level of SELENBP1 expression is reduced throughout the progression of pathogenesis of breast cancer, which is a later event and is also correlated with ER status.

In agreement with the report by Lai *et al* that SELENBP1 expression was largely decreased in the more malignant cell line MB231 but not in the less aggressive MCF7 [37], and ER^+^ and luminal breast cancer usually has a better prognosis than the ER^–^ and basal subtype breast cancer, we found that most ER^+^ and luminal breast cancer cells expressed high level of SELENBP1 but not ER^–^ and basal ones. Among luminal subtype of cells, most are ER^+^ cells except two cell lines MB 453 and SKBR3 which are ER^–^ and yet express high levels of SELENBP1. Analysis from previous publication [29] on breast cancer cell lines showed that these two cell types are all derived from adenocarcinoma but not invasive ductal carcinoma which is different from most cells that are both ER^–^ and basal subtype. This correlation is also in agreement with our finding that SELENBP1 high cell lines are less aggressive than SELENBP1 low cell lines regardless their ER status.

In ER^+^ breast cancer cells, we found that SELENBP1 expression was reduced upon exogenous estrogen treatment. Similar results were demonstrated in two previous reports [27], [28]. Yoshida and collaborators demonstrated that SELENBP1 was one of the estrogen responsive/regulated genes [28]. Real-time PCR data by Suzuki and collaborators showed that there was a statistically significant downregulated expression of SELENBP1 mRNA upon E2 treatment [27]. In our Fig. 5, we observed a trend of down-regulation of SELENBP1 protein upon 24 hours of estrogen treatment and the significant SELENBP1 reduction occurs in 48 hours of treatment. Analysis of SELENBP1 gene promoter region reveals three estrogen response elements (ERE), indicating a possible ER direct function on SELENBP1. Therefore, we speculate that ER may exert both direct and indirect functions on SELENBP1 expression. As estrogen regulates genes through MAPK, PI3K, and PKA signaling pathways, it may indirectly regulates SELENBP1 through other pathways, the tethered pathway which includes protein-protein interaction with other transcription factors after ligand activation, and thereby gene regulation is affected by indirect DNA binding. Further studies are warrant for decipher the details of SELENBP1 downregulation by estrogen. In ER^+^ and luminal breast cancer cells, pathological level of estrogen in serum and tissue fluids promote carcinogenesis and tumor growth by decreasing SELENBP1 expression, subsequently abolishing the anti-tumor effect of selenium. ER^–^ and basal cells of breast cancer are resistant to selenium-mediated effects on cancer because of little to no level of SELENBP1 expression. Thus, our data indicate that basal and luminal breast cancer cells that are resistant to the Selenium’s cancer prevention and treatment effects are most probably due to downregulated SELENBP1. Our study also indicates a novel mechanism through which estrogen could promote tumorigenesis in ER^+^ breast cells, by downregulating SELENBP1 through ER.

In accordance with previous reports on the resistance of ER^+^ breast cancer cells, an indirect study by Li and collaborators showed that combinations of selenium and tamoxifen inhibit growth of ER^+^ breast cancer cells MCF7 *in vivo* by promoting apoptosis [38]. Although we cannot exclude the fact that tamoxifen might have had more complicated effects than its role as an ER blocker, these results showed similar observation as shown in our study. We showed that estrogen treatment downregulates SELENBP1 expression in ER^+^ cells and therefore abolished the cell proliferation inhibition effect of Se treatment. But if ER was silenced by shRNA, estrogen treatment can no longer affect the cell proliferation inhibition effect exerted by Se treatment. These data indicating the level of SELENBP1 in breast cancer cells affects Se treated cell proliferation inhibition. It can be again proved in our Fig. 6B, although we used SELENBP1 specific shRNA to knock down SELENBP1, there are still residual SELENBP1 that cannot be completely eliminated by this technology, hence, we observe that inhibition of cell growth by Se is lower in the MCF7 SELENBP1 knock out cells as compared to control, but there is still some inhibition of growth by Se due to the remaining SELENBP1 level. Combined with previous finding and the results presented in our study, we believe that SELENBP1 reduced in ER^+^ cells might due to high estrogen levels, and that neutralizing the effect of estrogen in down-regulating SELENBP1 expression can enhance the function of selenium in cancer prevention and treatment.

In summary, we found that reduced SELENBP1 expression in breast cancer correlated with late stages of the disease and poor survival. High levels of SELENBP1 expression found in ER^+^ and luminal breast cancer cells can be downregulated by addition of exogenous estrogen. Patients with ER^+^ state tumor and low SELENBP1 had poorer survival rates. Decrease of SELENBP1 expression upon estrogen treatment or silencing SELENBP1 rendered ER^+^ breast cancer cells resistant to selenium treatment. Our data indicate that SELENBP1 could be an underestimated important marker for predicting patient survival, a marker to predict effective selenium prevention in breast cancer high risk patients and treatment in breast cancer patients. We also found that estrogen levels are another important factor for breast cancer prevention and treatment through a novel pathway involving SELENBP1 downregulation.

## Supporting Information

File S1 **Supporting figures.** Figure S1 Reduced SELENBP1 expression in hyperplastic enlarged lobular analyzed from public microarray database. The raw data from the microarray study were downloaded from NCBI Gene Expression Omnibus (GEO) or EBI ARRAYEXPRESS. The gene information and annotation of data sets were downloaded from the manufacturer of the microarray. The raw data and array information were inputted into dchip analysis software. After normalization and modeling, expression values (mean fluorescent intensity) were exported. Statistical analysis and boxplot graph were performed in SPSS software. *denotes p<0.05. The level of SELENBP1 expression is shown in 8 paired (16 total cases) of normal terminal and hyperplastic enlarged breast lobular cells. Figure S2 Differential levels of SELENBP1 expression in breast cancer cell lines analyzed from public microarray database. Microarray data analysis was performed as described previously. The level of SELENBP1 expression is shown in (A) microarray data for 12 breast cancer cell lines from GEO (GSE12777) (B) microarray data for12 breast cancer cell lines from ARRAYEXPRESS (E-TABM-157). Figure S3 Determination of ER knock-down by ERα-specific siRNA. ER^+^ MCF7 cells were transfected with either scrambled control or ERα-specific siRNA duplexes by Lipofectamine 2000. ER expression levels were determined by western blot at 48 h after transfection. Figure S4 Downregulation of SELENBP1 expression in MCF-7 cells upon estrogen treatments analyzed from public microarray database. Microarray data analysis was performed as described previously. (A) A time-dependent reduction of SELENBP1 expression upon E2 treatment. Dunnett T3 test was performed to show p values compared with each time point. The data sets are from GSE11324. (B). Reduction of SELENBP1 expression up to 48 h of E2 treatment. The data sets are from GSE11352. Figure S5 Determination of ER expression after ER plasmid transfection. ER^–^ SKBR3 and MDAMB453 cell lines were transfected with ER expression plasmid using Lipofectamine 2000. Cell lysates were collected at different time points indicated to check ER expression by western blot. Beta-actin was used as an equal loading control. MCF7 cells were used as a positive control. Figure S6 Determination of SELENBP1 expression levels by overexpression or knock-out SELENBP1 plasmids in breast cancer cells. MCF7 cells were transfected with shRNA vector control, or Non-effective 29-mer scrambled shRNA control, or Human SELENBP1-specific shRNA, at 72 h post transfection, cell lysates were collected to proceed for western blot to determine SELENBP1 expression. MDAMB231 cells were transfected with either vector control or SELENBP1 overexpression plasmid. At 48 h of transfection, cell lysates were collected and SELENBP1 expression level was determined by western blot.
(PPT)
