# Supplementary material for: Reduced Selenium-Binding Protein 1 in Breast Cancer Correlates with Poor Survival and Resistance to the Anti-Proliferative Effects of Selenium
Source: PLoS One. 2013 May 21;8(5):e63702. doi: 10.1371/journal.pone.0063702 (PMC3660592; doi:10.1371/journal.pone.0063702)
Supplement: File S1 — Supporting figures. Figure S1 Reduced SELENBP1 expression in hyperplastic enlarged lobular analyzed from public microarray database. The raw data from the microarray study were downloaded from NCBI Gene Expression Omnibus (GEO) or EBI ARRAYEXPRESS. The gene information and annotation of data sets were downloaded from the manufacturer of the microarray. The raw data and array information were inputted into dchip analysis software. After normalization and modeling, expression values (mean fluorescent intensity) were exported. Statistical analysis and boxplot graph were performed in SPSS software. *denotes p<0.05. The level of SELENBP1 expression is shown in 8 paired (16 total cases) of normal terminal and hyperplastic enlarged breast lobular cells. Figure S2 Differential levels of SELENBP1 expression in breast cancer cell lines analyzed from public microarray database. Microarray data analysis was performed as described previously. The level of SELENBP1 expression is shown in (A) microarray data for 12 breast cancer cell lines from GEO (GSE12777) (B) microarray data for12 breast cancer cell lines from ARRAYEXPRESS (E-TABM-157). Figure S3 Determination of ER knock-down by ERα-specific siRNA. ER+ MCF7 cells were transfected with either scrambled control or ERα-specific siRNA duplexes by Lipofectamine 2000. ER expression levels were determined by western blot at 48 h after transfection. Figure S4 Downregulation of SELENBP1 expression in MCF-7 cells upon estrogen treatments analyzed from public microarray database. Microarray data analysis was performed as described previously. (A) A time-dependent reduction of SELENBP1 expression upon E2 treatment. Dunnett T3 test was performed to show p values compared with each time point. The data sets are from GSE11324. (B). Reduction of SELENBP1 expression up to 48 h of E2 treatment. The data sets are from GSE11352. Figure S5 Determination of ER expression after ER plasmid transfection. ER– SKBR3 and MDAMB453 cell lines were transfe [file pone.0063702.s001.ppt]

## Slide 1
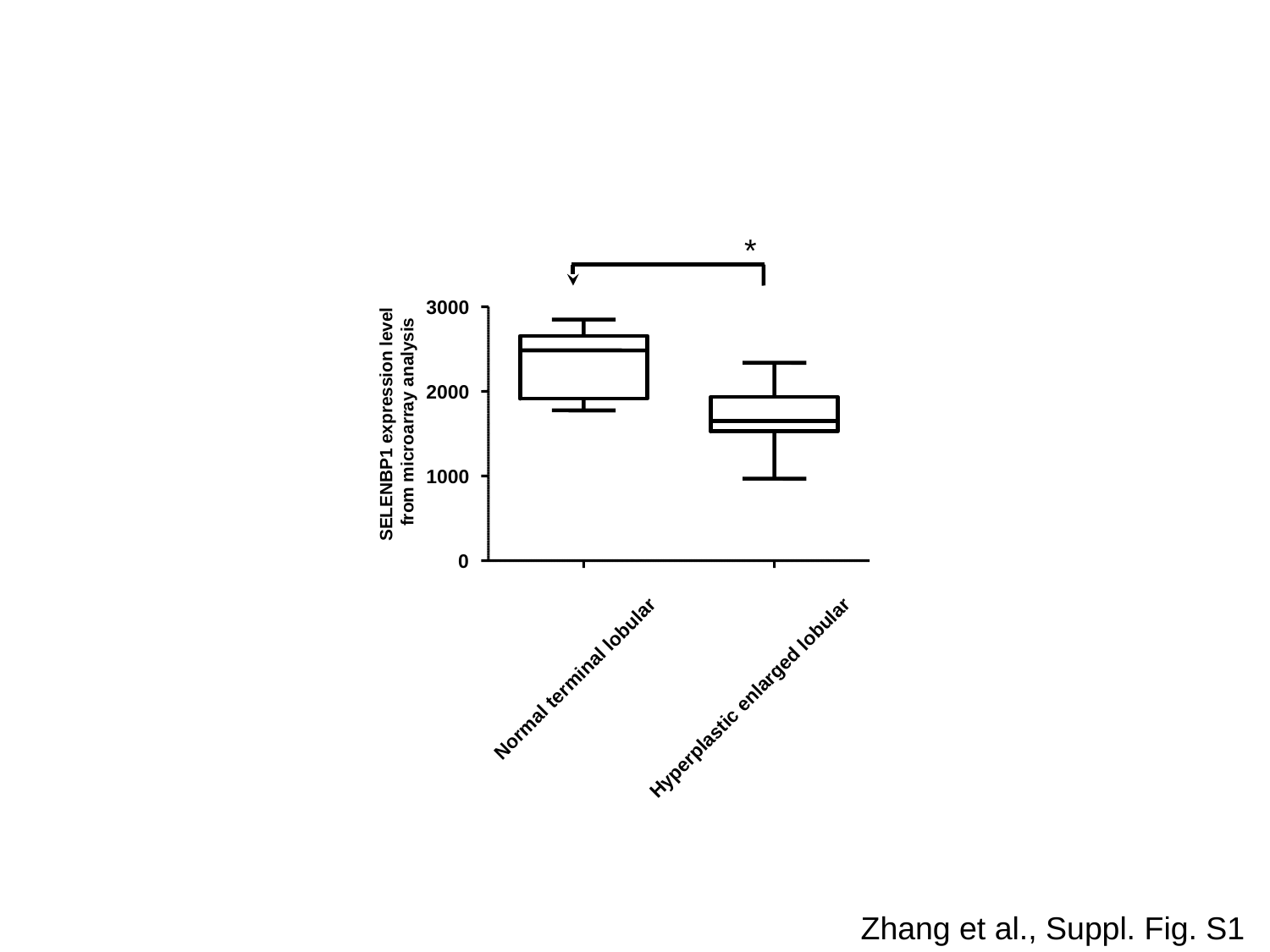

*
3000
2000
SELENBP1 expression level
from microarray analysis
1000
0
Normal terminal lobular
Hyperplastic enlarged lobular
Zhang et al., Suppl. Fig. S1

## Slide 2
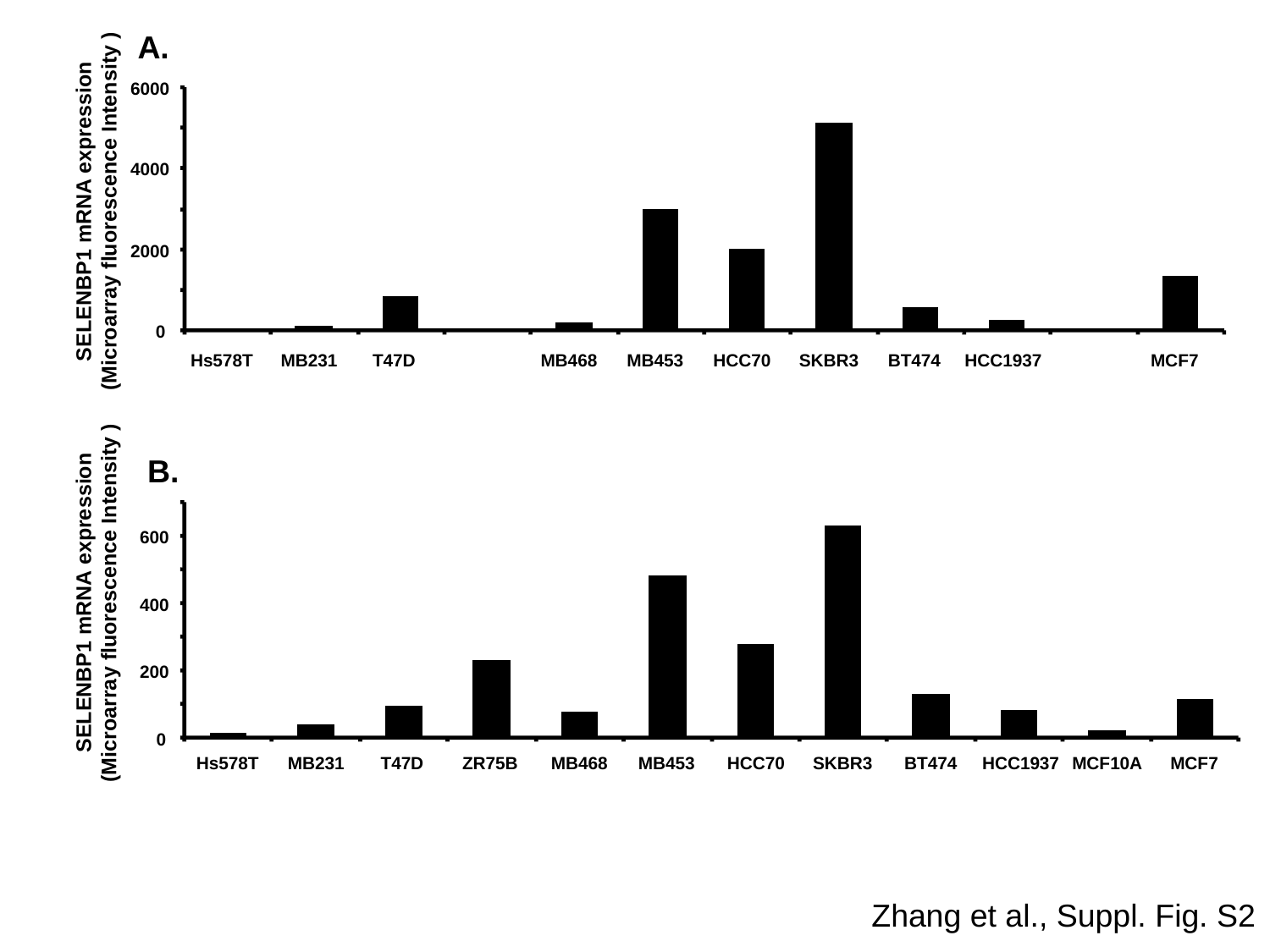

A.
6000
4000
SELENBP1 mRNA expression (Microarray fluorescence Intensity )
2000
0
Hs578T
MB231
T47D
MB468
MB453
HCC70
SKBR3
BT474
HCC1937
MCF7
B.
600
400
200
0
Hs578T
MB231
T47D
ZR75B
MB468
MB453
HCC70
SKBR3
BT474
HCC1937
MCF10A
MCF7
SELENBP1 mRNA expression (Microarray fluorescence Intensity )
Zhang et al., Suppl. Fig. S2

## Slide 3
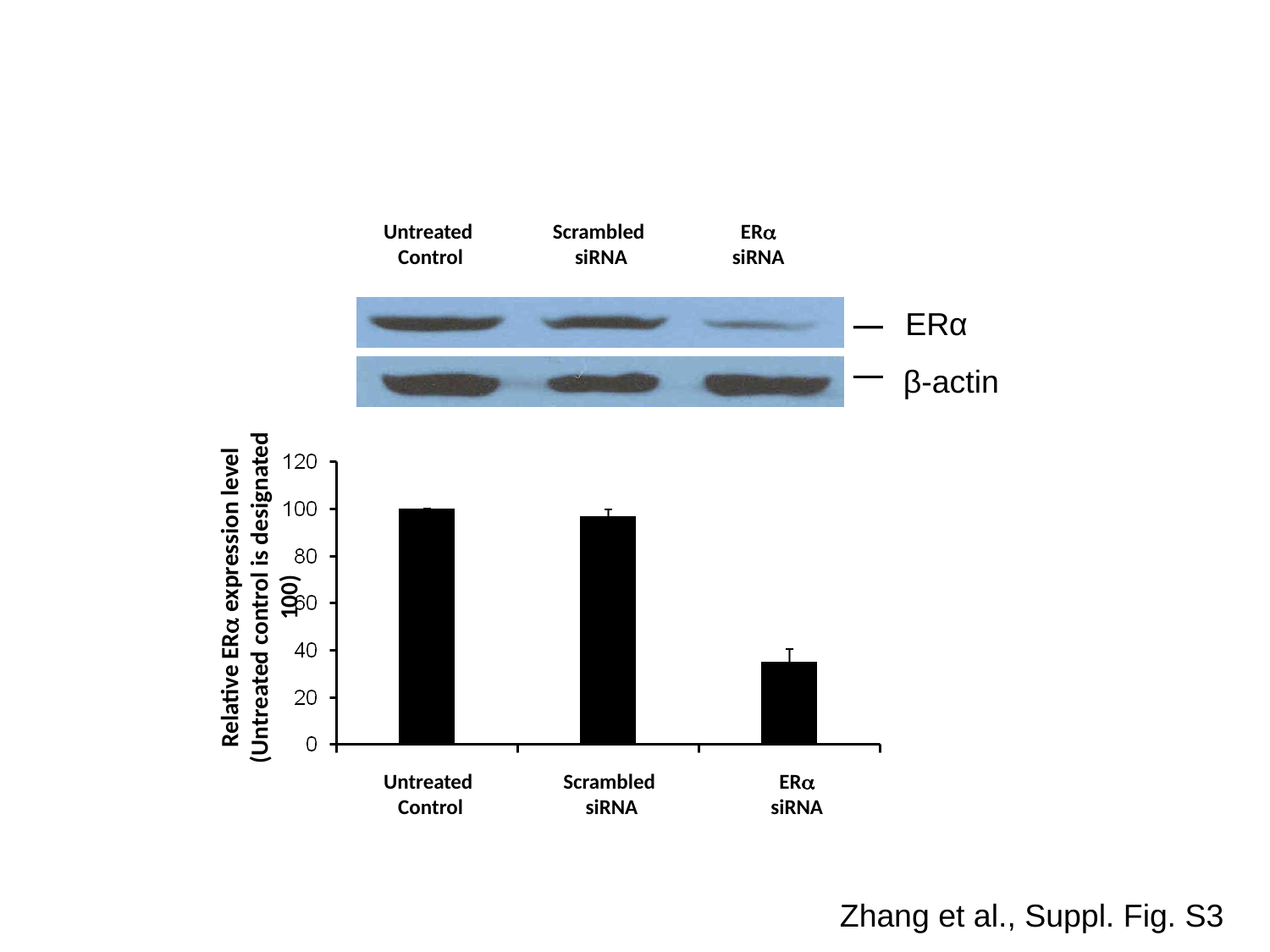

Untreated
Control
Scrambled
siRNA
ERα
siRNA
ERα
β-actin
Relative ERα expression level
(Untreated control is designated 100)
Untreated
Control
Scrambled
siRNA
ERα
siRNA
Zhang et al., Suppl. Fig. S3

## Slide 4
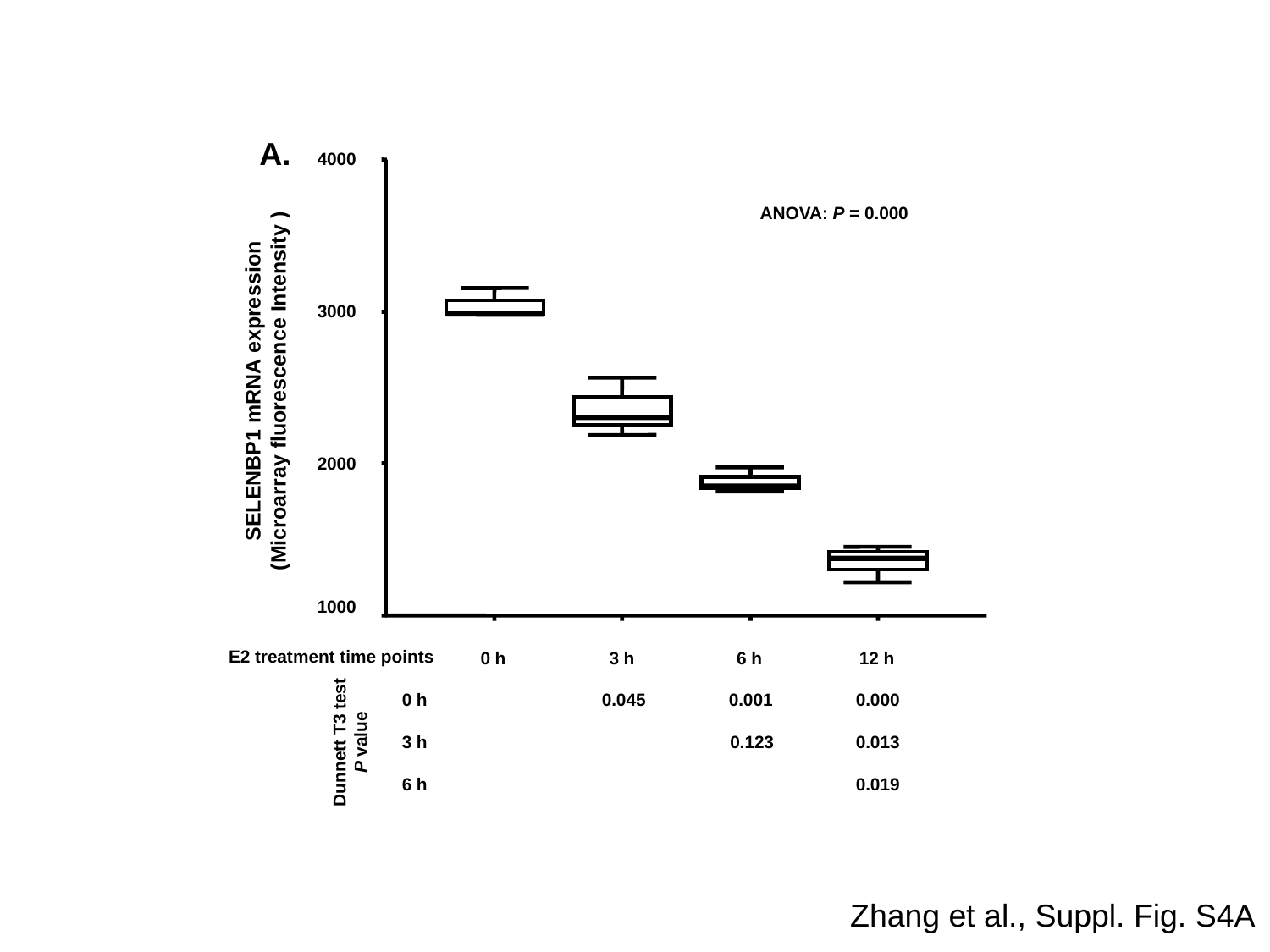

A.
4000
ANOVA: P = 0.000
3000
SELENBP1 mRNA expression (Microarray fluorescence Intensity )
2000
1000
E2 treatment time points
0 h
3 h
6 h
12 h
0 h
0.045
0.001
0.000
Dunnett T3 test
P value
3 h
0.123
0.013
6 h
0.019
Zhang et al., Suppl. Fig. S4A

## Slide 5
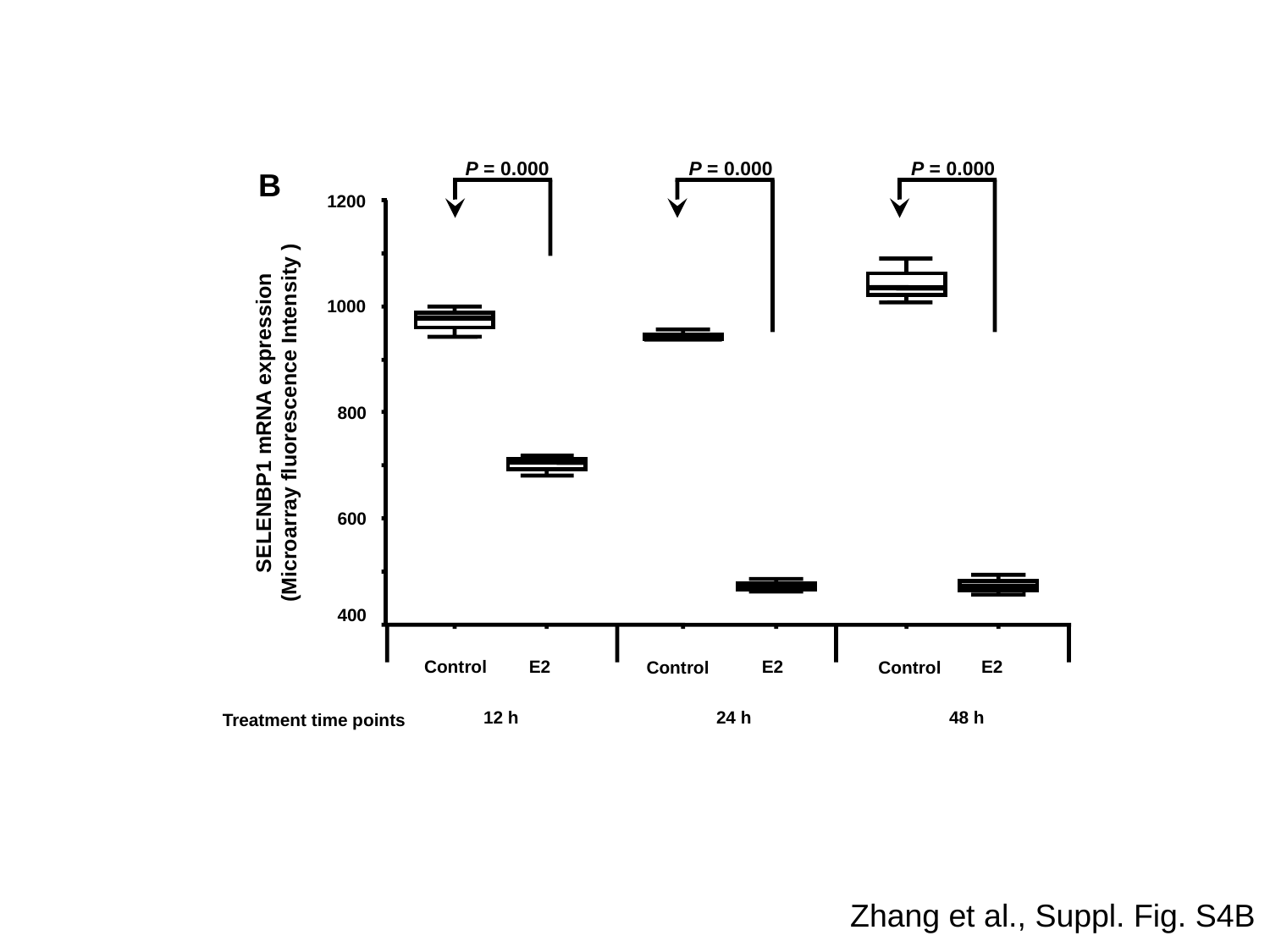

P = 0.000
P = 0.000
P = 0.000
B
1200
1000
SELENBP1 mRNA expression (Microarray fluorescence Intensity )
800
600
400
Control
E2
E2
E2
Control
Control
12 h
24 h
48 h
Treatment time points
Zhang et al., Suppl. Fig. S4B

## Slide 6
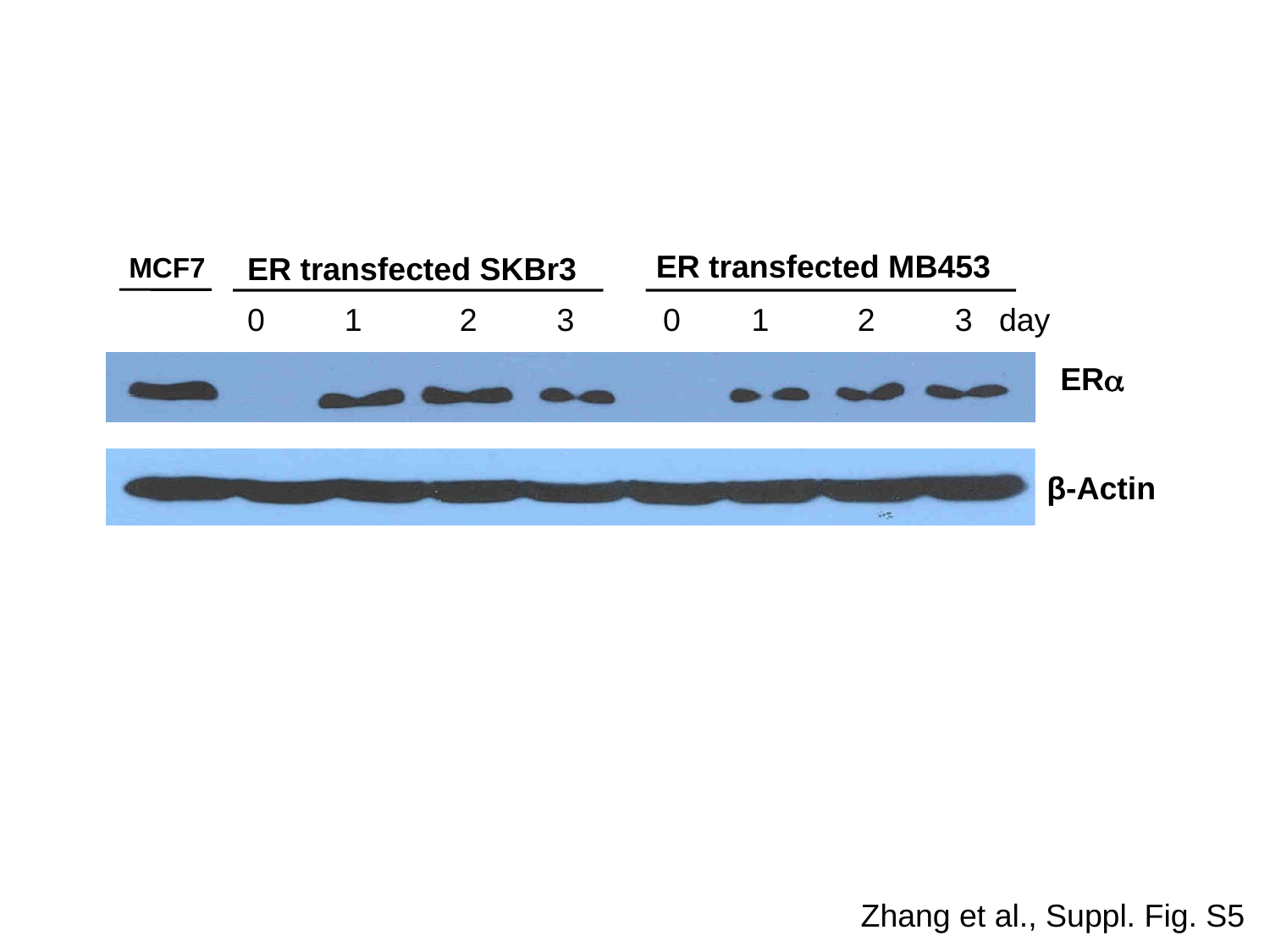

ER transfected MB453
ER transfected SKBr3
MCF7
0 1 2 3 0 1 2 3 day
ERα
β-Actin
Zhang et al., Suppl. Fig. S5

## Slide 7
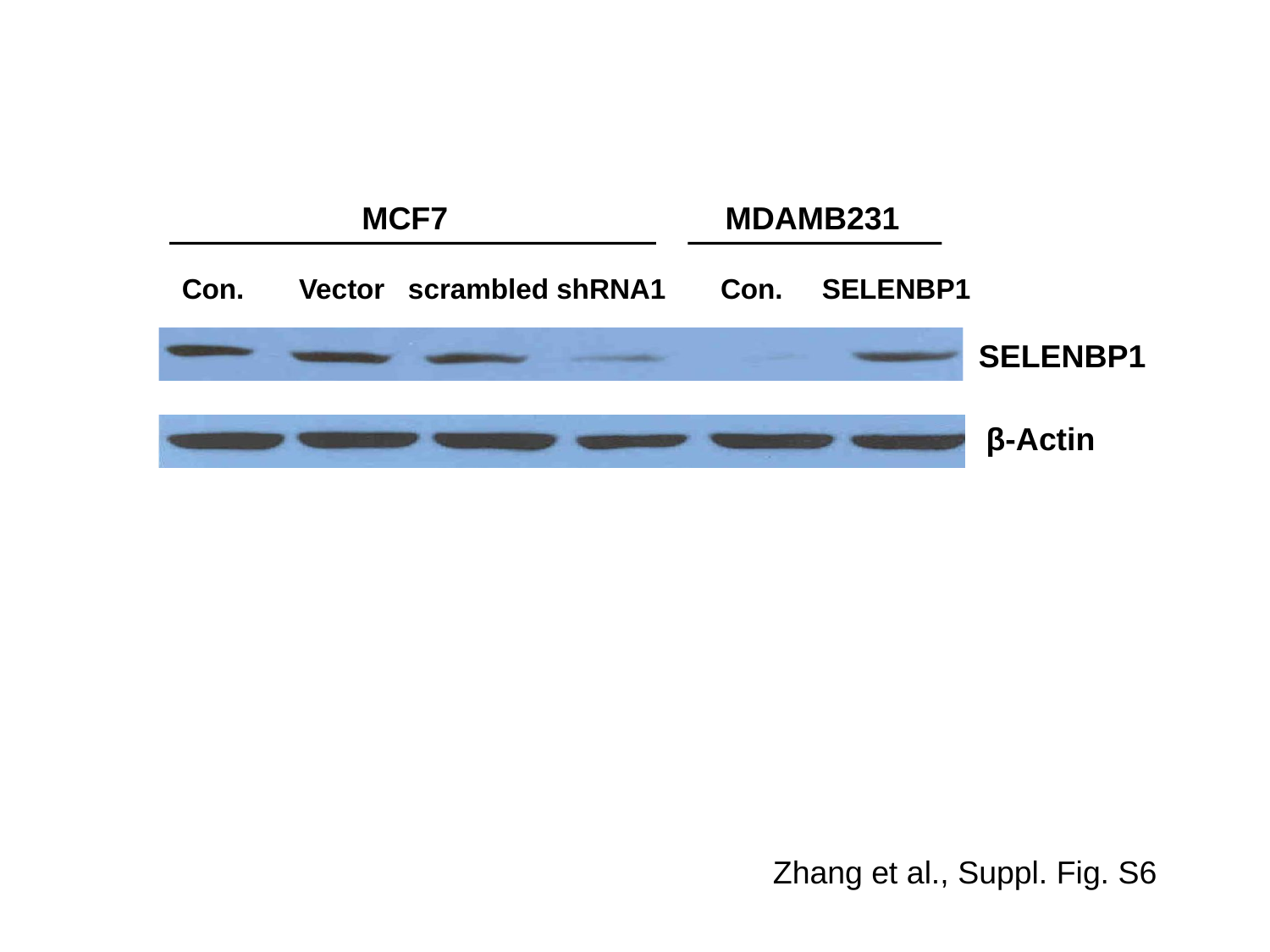

MCF7
MDAMB231
Con. Vector scrambled shRNA1 Con. SELENBP1
SELENBP1
β-Actin
Zhang et al., Suppl. Fig. S6
